# Motor cortical inhibitory deficits in patients with obsessive-compulsive disorder–A systematic review and meta-analysis of transcranial magnetic stimulation literature

**DOI:** 10.3389/fpsyt.2022.1050480

**Published:** 2022-12-07

**Authors:** Daniel Rodrigues da Silva, Ana Maia, Gonçalo Cotovio, José Oliveira, Albino J. Oliveira-Maia, J. Bernardo Barahona-Corrêa

**Affiliations:** ^1^Champalimaud Research and Clinical Centre, Champalimaud Foundation, Lisbon, Portugal; ^2^NOVA Medical School, NMS, Universidade Nova de Lisboa, Lisbon, Portugal; ^3^Department of Psychiatry and Mental Health, Centro Hospitalar de Lisboa Ocidental, Lisbon, Portugal

**Keywords:** obsessive-compulsive disorder (OCD), transcranial magnetic stimulation, corticospinal excitability, systematic review, meta-analysis

## Abstract

**Introduction:**

Obsessive-compulsive disorder (OCD) is a highly prevalent chronic disorder, often refractory to treatment. While remaining elusive, a full understanding of the pathophysiology of OCD is crucial to optimize treatment. Transcranial magnetic stimulation (TMS) is a non-invasive technique that, paired with other neurophysiological techniques, such as electromyography, allows for *in vivo* assessment of human corticospinal neurophysiology. It has been used in clinical populations, including comparisons of patients with OCD and control volunteers. Results are often contradictory, and it is unclear if such measures change after treatment. Here we summarize research comparing corticospinal excitability between patients with OCD and control volunteers, and explore the effects of treatment with repetitive TMS (rTMS) on these excitability measures.

**Methods:**

We conducted a systematic review and meta-analysis of case-control studies comparing various motor cortical excitability measures in patients with OCD and control volunteers. Whenever possible, we meta-analyzed motor cortical excitability changes after rTMS treatment.

**Results:**

From 1,282 articles, 17 reporting motor cortex excitability measures were included in quantitative analyses. Meta-analysis regarding cortical silent period shows inhibitory deficits in patients with OCD, when compared to control volunteers. We found no statistically significant differences in the remaining meta-analyses, and no evidence, in patients with OCD, of pre- to post-rTMS changes in resting motor threshold, the only excitability measure for which longitudinal data were reported.

**Discussion:**

Our work suggests an inhibitory deficit of motor cortex excitability in patients with OCD when compared to control volunteers. Cortical silent period is believed to reflect activity of GABA_B_ receptors, which is in line with neuroimaging research, showing GABAergic deficits in patients with OCD. Regardless of its effect on OCD symptoms, rTMS apparently does not modify Resting Motor Threshold, possibly because this measure reflects glutamatergic synaptic transmission, while rTMS is believed to mainly influence GABAergic function. Our meta-analyses are limited by the small number of studies included, and their methodological heterogeneity. Nonetheless, cortical silent period is a reliable and easily implementable measurement to assess neurophysiology in humans, *in vivo*. The present review illustrates the importance of pursuing the study of OCD pathophysiology using cortical silent period and other easily accessible, non-invasive measures of cortical excitability.

**Systematic review registration:**

[https://www.crd.york.ac.uk/prospero/display_record.php?ID=CRD42020201764], identifier [CRD42020201764].

## Introduction

Obsessive-compulsive disorder (OCD) is a chronic and highly incapacitating neuropsychiatric disorder with a lifetime prevalence of 1.3%, and is a major contributor to the health-economic burden of mental disorders (1, 2). First-line treatments include pharmacotherapy, typically with a serotonin reuptake inhibitor, cognitive-behavioral psychotherapy (CBT), or a combination of both. Between 40 and 60% of patients with OCD fail to achieve response criteria with first-line treatments, advising for the need to improve our knowledge regarding therapeutic options for this clinical condition (3). New neuromodulatory approaches to treatment, namely, transcranial magnetic stimulation (TMS) (4) and deep brain stimulation (DBS) (5) offer novel possibilities for treatment-resistant OCD. Several targets and stimulation parameters have been tested for the treatment of OCD using TMS, but no clear consensus exists regarding the best combination of stimulation parameters and stimulation target. While several studies support low-frequency protocols over the dorsolateral prefrontal cortex (6), others found greater effectiveness for low frequency rTMS over the supplementary motor area or for specific combinations of different targets and stimulation parameters (7, 8). All these studies have used rTMS delivered with a traditional figure-of-eight coil. In 2019 deep TMS (dTMS), delivered at high frequencies over the medial prefrontal cortex with a coil specially designed to reach deeper structures in the brain, received FDA clearance for the treatment of OCD, based on the work of Carmi et al. (4). Despite these unquestionable advances, optimization of treatment remains critically dependent on our ability to fully understand the pathophysiology of this complex disorder, as well as our ability to develop reliable predictors of treatment response at an individual level (9).

While much of the pathophysiology of OCD remains a mystery, there is reasonable consensus that it involves dysfunction of cortico-striato-thalamo-cortical (CSTC) circuits underlying sensorimotor, cognitive, affective, and motivational processes (10). Additionally, imaging studies comparing patients with OCD and control volunteers consistently report increased volumes of putamen, cerebellum and striatum (11, 12). This is in contrast with smaller volumes of the dorsomedial prefrontal, medial orbitofrontal and insular opercular cortices and hippocampus, which also are reported in studies comparing patients with OCD and control volunteers (13–15). With regards to neurochemical mechanisms, there is evidence that OCD may be associated with impaired functioning of major neurotransmitter systems, such as serotonin, dopamine, glutamate, and GABA. Such evidence is mostly indirect and relies on measurements performed with MRI spectroscopy or neurophysiological techniques (16–19). One such technique is TMS, typically coupled with electromyography (EMG). TMS is a non-invasive brain stimulation technique based on electromagnetic induction. Through transmission of an intense, brief pulse of electrical current through loops of wire, pulsatile magnetic fields are generated perpendicularly to the plane of the coil. These magnetic fields penetrate the scalp and skull adjacent to the coil, inducing electric gradients in cortical tissue that can modify neuronal activity (20). When paired with EMG as a way of quantitatively assessing motor evoked potentials induced by TMS pulses, TMS allows for the assessment of various measures of cortical responsiveness that are considered indirect measures of specific neurotransmitter function, such as cortical silent period (believed to reflect GABA_B_ receptor-mediated inhibition), short-interval intracortical inhibition (believed to reflect GABA_A_ receptor mediated inhibition), or intracortical facilitation (possibly reflective of glutamate-mediated excitatory interneuronal circuits) (21–25). In the specific case of OCD, this approach has been used to compare measures of cortical excitability in patients with OCD and control volunteers. Unfortunately, studies have adopted a wide diversity of TMS-EMG experimental paradigms, and this has led to inconsistent and sometimes contradictory results (26–29).

To the best of our knowledge, there have been no systematic reviews specifically dedicated to studies using TMS-EMG paradigms to assess cortical excitability in OCD, although a 2013 review and meta-analysis of cortical excitability abnormalities in various neuropsychiatric disorders included two studies conducted in patients with OCD (30). Here we propose to fill that gap by systematically reviewing studies that used TMS-EMG paradigms to assess corticospinal excitability in patients with OCD and control volunteers, and performing meta-analyses and meta-regressions whenever possible. Our main focus will be case-control differences in motor cortical excitability. Secondarily, in an exploratory approach, we will assess the association between treatment of OCD with rTMS and changes in motor cortical excitability.

## Materials and methods

### Protocol and registration

The systematic review protocol is published in the PROSPERO database (CRD42020201764) and is freely available to consult at https://www.crd.york.ac.uk/prospero/display_record.php?ID=CRD42020201764.

### Information sources and search strategy

The systematic literature search was performed on EMBASE, Web-of-Science, PubMed, and PsycINFO databases. Our search considered papers published until March 2022. Syntax was as follows, considering adaptations based on rules established by each search database: diagnosis of interest (obsessive-compulsive disorder, OCD, obsession/obsessive symptoms, compulsion/compulsive symptoms, anxiety disorders) and neurophysiology-related terms (theta burst stimulation, TBS, transcranial magnetic stimulation, TMS, excitation, excitability, modulation, modulate, control, change, modify, activity, activate, deactivate, facilitate, facilitation, inhibit, improve, impair, inhibition, adjust, adjustment, transform, induce, modulated, decrease, affect). Study selection was filtered based on study model and language, with only studies with human subjects reported in English, Portuguese, Spanish, French, or German considered. No filters were applied regarding publication date or country of origin (Supplementary Method 1).

### Study selection and eligibility criteria

After the removal of duplicate entries, two researchers (DRS and AM) independently reviewed the final list of eligible articles, proceeding to its filtering based on PRISMA guidelines (31). To be considered eligible for synthesis, studies needed to have a case-control design evaluating measures of motor cortical excitability in patients with OCD, diagnosed according to the Diagnostic and Statistical Manual of Mental Disorders (DSM-III or later edition), or its equivalent in the International Classification of Diseases (ICD-9 or later editions), and also in control volunteers. Studies without a control-group of healthy subjects were retained for exploratory analyses addressing specific questions such as the association between motor cortical excitability and disease severity (meta-regression), or the association between treatment with rTMS and longitudinal within-subject changes in motor cortical excitability measures (longitudinal meta-analysis). To perform such analysis, we included the active arm of randomized controlled-trials, open-label studies, cohort studies and case series with a minimum of three subjects. Study eligibility was also dependent on the acquisition of at least one type of motor cortical excitability measure. Specifically, the following excitability measures were considered: resting or active motor threshold (RMT/AMT); cortical silent period (CSP); motor evoked potential amplitude, latency or area under the curve (MEP); 140/120 ratio–motor evoked potential amplitude ratio at increasing stimulus intensity from 140 to 120%; intra-cortical facilitation (ICF); short-interval intracortical inhibition (SICI); long-interval intra-cortical inhibition (LICI); ratio and/or difference between all aforementioned excitability measures before and after a single session of rTMS or theta burst stimulation (TBS) or other excitability modulation protocol applied to motor cortex (i.e., excitability modulation measures). (For full description of each measure, please see Supplementary Table 1).

Studies were deemed ineligible if there was no formal OCD diagnosis, or if they included individuals diagnosed with a major central nervous system (CNS) disorder–e.g., epilepsy, multiple sclerosis, amyotrophic lateral sclerosis, Parkinson’s disease, Huntington’s disease; individuals with cancer with known CNS involvement, or with co-morbid major peripheral nervous system (PNS), neuromuscular system (NMS) or muscle-skeleton system (MSS) disorders–e.g., Guillain-Barré syndrome, muscular dystrophias; and individuals with other medical conditions that may influence the CNS, PNS, NMS, or MSS – e.g., hepatic failure, paraneoplastic syndrome, chronic, or acute renal failure, heart failure or other severely debilitating cardiovascular conditions, uncontrolled diabetes. Studies without peer-review, case reports (or case series with less than three patients), literature reviews and meta-analyses were also not included in the final list of included studies. However, the reference lists of these publications were searched for additional eligible references that might have escaped the original search strategy. Reference lists of all the included studies were likewise screened for the same purpose.

### Data extraction, data items and risk of bias

Two researchers (DRS and AM) extracted data separately, according to PRISMA guidelines (31). The following variables were collected: first author name, year of publication, type of study, title, publication journal, sample sizes in total and per group (i.e., clinical and control volunteer groups). For each group we extracted data regarding handedness, sex, age, OCD age of onset and/or illness duration, treatment refractoriness, severity of the disorder (as assessed by formal psychometric instruments at baseline and post-treatment, if applicable). We also included TMS-related variables, namely, stimulation frequency, stimulation intensity and motor cortical excitability measures and their respective method of acquisition. Whenever available, we extracted data for RMT, AMT, MEPs, SICI, LICI, ICF, CSP, 140/120 ratio, and the ratio and/or change in the previously mentioned measures after a single session of rTMS or TBS. When needed, corresponding author of eligible studies were contacted to request additional data or clarify information provided in the articles. Study quality score was established by consensus between DRS and AM, according to the Newcastle-Ottawa Quality Assessment Scale (NOS) for case-control studies (32).

### Data analysis

Analyses were performed using *Rstudio’s meta* package (33) and *Comprehensive Meta-analysis*, Version 3. SPPS, version 26 (*IBM* corp.) was used to compute means, standard deviations, and other metrics, when individual data was provided by contacted authors. For our primary goal, we conducted random effects model pairwise meta-analyses of individual motor cortical excitability measures comparing patients with OCD and control volunteers. Hedge’s *g* effect size estimates were computed using *metacont* (33) function (*Rstudio*), based on sample size, mean, and standard deviation provided in each study for each individual excitability measure, both for patients with OCD and control volunteers. While separate meta-analyses were conducted for each excitability measure individually, whenever a study reported more than one measurement of the same excitability measure, these results were averaged (34). For instance, if a CSP measurement was conducted both at 110 and 120% of the RMT and authors report results separately for each assessment, an average of these two results was calculated and entered into the meta-analysis. The same approach was used when SICI and ICF measures were reported at different interstimulus intervals (ISI) in the same study.

For exploratory purposes, active arms of controlled studies were analyzed jointly with open-label studies, cohort studies and case series with a minimum of three subjects. Data from these studies were entered into an exploratory random effects model pairwise meta-analysis of change in motor cortical excitability from before to after a treatment. Hedge’s *g* effect size estimates were computed based on the formula proposed by Borenstein et al. (35) to correct effect size and variance in pre-post design studies. Mean within-group differences between pre- and post-treatment cortical excitability were likewise computed according to Borenstein et al. (35) (For a full description, please see Supplementary Methods 2, 3). Meta-analyses based on such effect sizes and respective *SE* were computed with *RStudio’s metagen* function, using an inverse variance method (33).

Univariate meta-regression models were conducted to assess variables that could moderate motor cortical excitability values in patients with OCD, when at least 10 studies were available (34). Per-protocol, we were interested in the following moderators for univariate meta-regression models: age, sex, handedness, age at onset of OCD, baseline severity scores, use of any medication or other treatment strategies, degree of refractoriness, TMS coil-type, pulse-type, stimulation frequency, stimulation intensity, and bias score (based on the NOS for the quality assessment of non-randomized articles).

Sensitivity analyses were performed for individual meta-analyses of each cortical excitability measure by restricting analyses to: papers with a NOS score <5; studies that used the same method to assess the motor cortical excitability measure of interest; intervention studies that found significant pre to post-rTMS reduction of OCD symptoms; intervention studies that used the same treatment protocol and parameters. We were also interested in assessing evidence of interhemispheric differences in motor cortical excitability in patients with OCD. Sensitivity analyses were only performed when a minimum of four studies remained in the restricted set of studies.

To assess heterogeneity, we estimated *I*^2^ and Egger et al. (36) method to assess publication bias (36), whenever ten or more papers were included in the meta-analysis. Otherwise, a significant risk of bias was automatically assumed.

## Results

### Literature review

**FIGURE 1:**
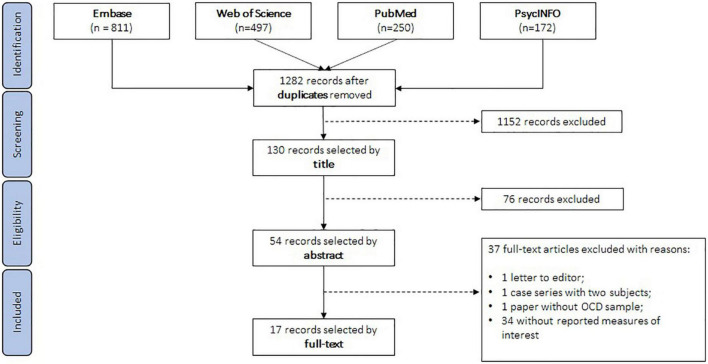
Study selection flowchart.

Our systematic search generated, after removal of duplicates, 1,282 entries. After an iterative review by title, abstract and full-text, 17 studies reporting motor cortex excitability measures and fulfilling all the criteria previously described were considered eligible for quantitative synthesis (Figure 1) (4, 26–29, 37–48). All 17 studies included patients with OCD, and 8 of them were case-control studies also including a comparison group of control volunteers (26–29, 38, 46–48). Only these 8 case-control studies were included in the primary analyses. Of the remaining 9 studies, 8 were randomized controlled trials, or open label studies (4, 37, 39–41, 43–45). One study was a case series with 14 participants (42). The most frequently reported measure was RMT, reported in all 17 studies (see Table 1 for full description of the studies and Supplementary Table 2 for additional details on cortical excitability measure results).

**TABLE 1:** Summary results of eligible studies.

References	Study design	Sample size			Active TMS treatment	Sham TMS treatment	Age (years: mean ± SD)		Female (%)	OCD age of onset (years: mean ± SD)	OCD symptom severity (YBOCS: mean ± SD)	Motor cortical excitability measures assessed					
									
		Total	OCD	CV			OCD	CV				R M T	A M T	M E P	I C F	S I C I	C S P
de Wit et al. (38)	Case control	77	39	38	–	–	39.13 ± 10.06	39.19 ± 11.44	51.95	–	21.26 ± 6.32	✓	–	–	–	–	–
Greenberg et al. (26)	Case control	27	16	11	–	–	38.1 ± 12.80	38.60 ± 13.90	37.40	–	14.60 ± 9.50	✓	✓	–	✓	✓	✓
Kang et al. (28)	Case control	90	51	39	–	–	27.43 ± 7.64	27.36 ± 6.99	22.22	15.90 ± 5.80	23.51 ± 7.23	✓	–	–	✓	✓	✓
Khedr et al. (29)	Case control	60	45	15	–	–	27.1 ± 4.50	28.30 ± 4.20	38.33	–	-	✓	✓	✓	✓	✓	✓
Mehta et al. (46)	Case control	168	43	125	–	–	29.2 ± 7.93	30.70 ± 7.52	31.23	–	26.61 ± 4.70	✓	–	–	–	✓	✓
Richter et al. (27)	Case control	68	34	34	–	–	40.94 ± 12.38	40.41 ± 10.26	54.41	–	24.34 ± 6.32	✓	–	✓	✓	✓	✓
Russo et al. (47)	Case control	24	12	12	–	–	30.20 ± 4.00	32.00 ± 20.00	41.67	–	–	✓	–	–	✓	✓	–
Suppa et al. (48)	Case control	35	15	20	-	-	32.3 ± 13.10	32.80 ± 11.20	28.57	–	23.80 ± 11.00	✓		–	–	–	–
	RCT	60	60	–	40	20	27.53 ± 6.37	–	51.67	–	23.82 ± 4.51	✓	–	–	–	–	–
Carmi et al. (4)	RCT	94	94	–	47	47	38.8 ± 11.84	–	38.80	13.13 ± 6.53	27.30 ± 4.00	✓	–	–	–	–	–
Donse et al. (39)	Open label	51	51	–	51		37.12 ± 12.49	–	46.50	–	26.88 ± 5.52	✓	–	–	–	–	–
Elbeh et al. (40)	RCT	45	45	–	30	15	27.85 ± 4.64	–	33.33	–	26.00 ± 5.62	✓		–	–	–	–
Harika-Germaneau et al. (41)	RCT	28	28	–	14	14	47.25 ± 11.41	–	53.57	22.10 ± 13.47	29.72 ± 4.47	✓	–	–	–	–	–
Hegde et al. (42)	Case series	14	14	–	14	–	27.86 ± 9.10	–	–	–	–	✓	–	–	–	–	–
Mantovani et al. (44)	Open Label	7	7	–	7	–	–	–	20.00	20.20 ± 1.90	36.40 ± 7.50	✓	–	–	–	–	–
Mantovani et al. (45)	RCT	18	18	–	9	9	39.55 ± 9.15	–	38.89	16.75 ± 8.96	26.35 ± 5.30	✓	✓	–	–	–	–
Kang et al. (43)	RCT	20	20	–	10	10	27.4 ± 11.39	–	15.00	18.75 ± 10.53	26.40 ± 4.78	✓	–	–	–	–	–
**Total**	–	878	558	320	222	115	33.3 ± 6.30	33.7 ± 4.50	38.30	17.8 ± 2.90	25.5 ± 4.60	–	–	–	–	–	–

OCD, obsessive-compulsive disorder; CV, control volunteers; YBOCS, Yale-Brown Obsessive Compulsive Scale; RMT, resting motor threshold; AMT, active motor threshold; MEP, motor evoked potential amplitude; ICF, intracortical facilitation; SICI, short-interval intracortical inhibition; CSP, cortical silent period; SD, standard deviation; RCT, randomized controlled trial.

The 17 eligible studies included in the review comprised a total of 878 participants, with 558 patients with OCD (*M* = 33.3; SD = 6.3 years old; 34.8% female) and 320 control volunteers (*M* = 33.7; SD = 4.5 years old; 41.8% female). Patients with OCD had a mean Yale-Brown Obsessive Compulsive Scale (Y-BOCS) score at enrollment of 25.5 (SD = 4.6). Age of onset for OCD was, on average, 17.8 (SD = 2.9) years old (Table 1). The 8 case-control studies included a total of 549 participants, with 255 patients with OCD and 294 control volunteers. Studies considered for the pre-post design meta-analyses comprised 120 patients with OCD (Table 1). Quality assessment of case-control studies rendered an average of 5.9 (SD = 1.9) obtained in the NOS scale (Supplementary Table 3).

### Results and synthesis of studies

**FIGURE 2:**
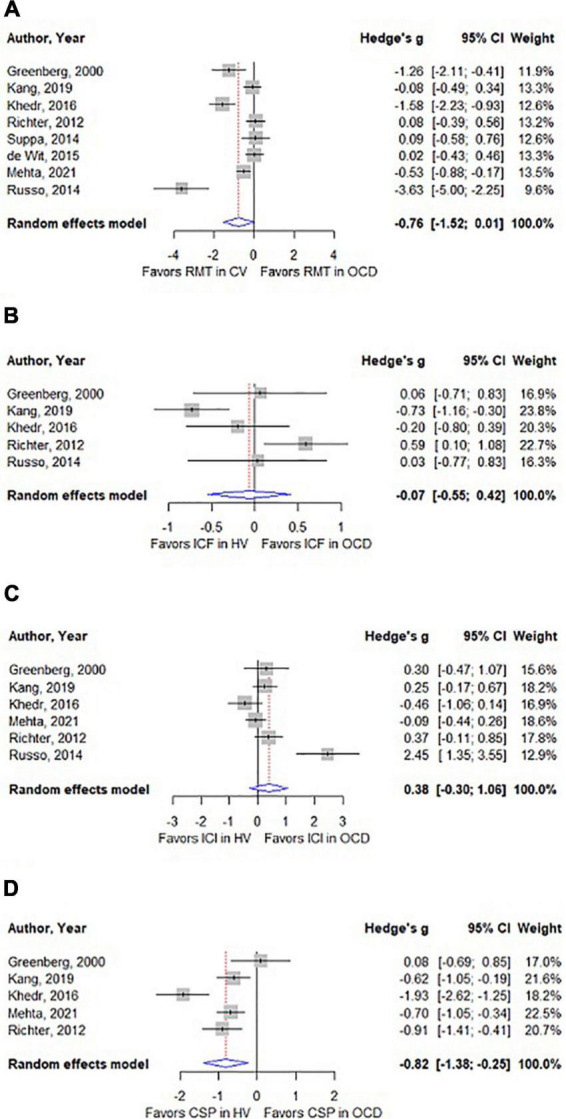
**(A)** Forest plot comparing resting motor threshold (RMT) between patients with OCD and control volunteers. **(B)** Forest plot comparing intracortical facilitation (ICF) between patients with OCD and control volunteers. **(C)** Forest plot comparing short intracortical inhibition (SICI) between patients with OCD and control volunteers. **(D)** Forest plot comparing cortical silent period (CSP) between patients with OCD and control volunteers.

Pairwise meta-analyses of motor cortical excitability measures comparing patients with OCD and control volunteers show that CSP is significantly shorter in patients with OCD [*N* = 5; g = −0.82; *p* < 0.01; *I*^2^ = 76.1%; Mean difference = −25.68 milliseconds; CI = (−40.45; −10.91)] (Figure 2D). For RMT the difference between patients with OCD and control subjects was not significant, albeit at a borderline level (*N* = 8; *g* = −0.76; *p* = 0.05; *I*^2^ = 86%) (Figure 2A). The other cortical excitability measures were not significantly different in patients with OCD and control volunteers: ICF (*N* = 5; *g* = −0.07; *p* = 0.78; *I*^2^ = 75.5%) (Figure 2B), and SICI (*N* = 6; *g* = 0.38; *p* = 0.27; *I*^2^ = 78.9%) (Figure 2C). For AMT and MEP amplitude no meta-analysis was performed due to a low number of includable studies (For data on these measures, please see Supplementary Tables 2, 4).

### Exploratory and sensitivity analyses

Exploratory meta-analyses of studies that assessed motor cortical excitability before and after rTMS treatment were only possible for left primary motor cortex RMT, for which pre- and post-TMS values were reported in five studies. Each of these five studies used a different treatment protocol (Supplementary Table 5). We found no significant change in RMT from pre- to post-treatment, in patients with OCD (*N* = 5; *g* = −0.03; *p* = 0.74; *I*^2^ = 0.0%) (Supplementary Figure 1 and Supplementary Table 6).

We performed further sensitivity analyses for RMT, SICI, ICF, CSP based on the NOS scores of the corresponding studies. Restricting analyses to studies with high NOS scores produced results that were no different from those of the main analyses, although the effect size for case-control differences in CSP was substantially larger in this restricted data-set (*N* = 4; *g* = −0.99; *p* < 0.001, *I*^2^ = 73.8%) (Supplementary Figure 2). The RMT meta-analysis was significant when we excluded two studies (38, 48) that measured RMT using a different methodology than the remaining studies (*N* = 6; *g* = −1.06; *p* = 0.03; *I*^2^ = 88.4%) (Supplementary Figure 3). Meta-analyses restricted to sham-controlled trials that reported a statistically superior clinical effect of rTMS treatment and of interhemispheric asymmetry in cortical excitability as measured by RMT among patients with OCD were not possible due to a low number of studies (For data on these measures, please see Supplementary Tables 4, 6). Additional sensitivity analyses based on homogeneity of the methods used to assess excitability measures were not possible due to the extensive methodologic heterogeneity (see Supplementary Table 7 for more detail on data acquisition).

### Meta-regressions

We only had enough data to perform univariate meta-regression models for RMT. We tested the association between this variable and age (*N* = 12; coefficient = 0.45; SE = 0.49; *p* = 0.36), sex (*N* = 13; coefficient = 0.22; SE = 0.19; *p* = 0.28), Y-BOCS scores (*N* = 12; coefficient = 0.08; SE = 0.60; *p* = 0.89), and percentage of medicated patients in each study (*N* = 13; coefficient = 0.03; SE = 0.09; *p* = 0.77). None of these moderators had a statistically significant effect on RMT.

## Discussion

Our study proposed to systematically review and to meta-analyze the existing literature on motor cortical excitability measured non-invasively using TMS-EMG paradigms, in OCD, and in control volunteers. We found limited evidence of increased motor cortical excitability in OCD compared to control volunteers, namely, a shorter duration of the CSP and, possibly, lower RMT.

Cortical silent period is a cortical excitability measure that reflects the duration of the period of suppression in electromyographic activity that typically follows a single TMS pulse delivered to the primary motor cortex while a mild voluntary contraction of the corresponding muscle of interest is being performed (49). It has good test-retest reliability under different conditions and methodological implementations (50, 51), making it a potentially useful marker of disease and/or treatment response for this clinical population. There is evidence that CSP reflects intracortical GABAergic activity, more specifically GABA_B_ receptor activity (52, 53), and our results are thus aligned with existing evidence that GABA_B_ receptor function may be impaired in OCD. In fact, patients with OCD tend to have lower plasma levels of GABA when compared to control volunteers (54). Furthermore, Magnetic Resonance Spectroscopy case-control studies in OCD have found lower concentrations of GABA-related metabolites in patients with OCD, predominantly in brain regions typically implicated in OCD’s pathophysiology, such as the orbitofrontal cortex (OFC) and the medial prefrontal cortex (mPFC) (55, 56). There is also evidence from genetic studies of an association between OCD susceptibility and certain polymorphisms of the *GABBR1* gene, responsible for the coding of GABA_B_ receptors (57). Finally, reduced activity of GABAergic inhibitory interneurons in the OFC has been described in an animal model of OCD, and correlates with learning deficits found in those animals (58).

The other measure for which we found evidence of increased cortical excitability in patients with OCD compared to the control group was RMT, although the difference was marginally non-significant and only became significant when we removed two studies from the analysis that measured RMT in an unconventional way. RMT is normally defined as the minimum intensity needed to elicit, at least, 5 muscle contractions or an electromyographic register of, at least, 50 μV, in a series of 10 pulses delivered to the primary motor cortex. This method was followed in the majority of the included studies. Our sensitivity analysis excluded those studies where a different method for the assessment of RMT was implemented. Meta-regression analyses showed that RMT in patients with OCD appears to be independent of age, sex, OCD symptom severity or medication. Left primary motor cortex RMT was also the only cortical excitability measure for which we had pre- to post-rTMS longitudinal data in a sufficient number of studies to explore whether rTMS treatment changes motor cortical excitability in OCD. We found no evidence that rTMS treatment of OCD results in a change of RMT. In fact, none of the five studies that report pre- and post-rTMS values of RMT found a significant change of this measure after treatment (Supplementary Figure 1), the notable exception being Montovani et al. (44) who found that low-frequency rTMS delivered to both right and left pre-motor supplementary motor areas increased RMT on the right, but not on the left primary motor cortex. Interestingly, effects on RMT appear to be independent of the effects of rTMS on OCD symptoms, since two of the four sham-controlled trials included in this group of studies found a significant decrease of Y-BOCS scores after rTMS treatment compared to sham (see data on these studies in Supplementary Tables 3, 4). A tentative explanation for this may be that RMT reflects neuronal membrane excitability and glutamatergic synaptic transmission, being unaffected by GABAergic agonists and antagonists (59, 60). Repetitive TMS is believed to affect mainly GABAergic transmission, and symptomatic improvement of OCD has been shown to correlate with an increase in GABA–but not glutamate–metabolites in the mPFC, as measured by proton magnetic resonance spectroscopy (61, 62).

None of the reviewed intervention studies explored to what degree cortical excitability measures at baseline are predictive of, or correlate with, symptomatic improvement after an rTMS protocol. This is in sharp contrast with the depression literature, where it has been shown that among patients with major depressive disorder those with lower RMT are more likely to respond to rTMS treatment for their condition (63), and lower RMT at treatment onset is predictive of a larger reduction in depression symptoms after 10 days of rTMS treatment (64). Such a knowledge gap In the OCD literature is unfortunate, since response to treatment is notoriously difficult to predict in OCD, being unsatisfactory or altogether absent in at least half of the treated patients. Finding easily accessible, reliable biomarkers of response to specific treatments such as rTMS, recently cleared as a treatment option for OCD, would undoubtedly contribute to improve our ability to personalize treatment and thus optimize the chances of meaningful improvement for individual patients. This is particularly important if we consider that treatment trials for patients with OCD are exceptionally long, with clinicians typically waiting 2 to 3 months to assess efficacy (65).

In contrast with our main finding, studies that combined TMS and EEG-derived measures of cortical excitability found no differences in cortical excitability between patients with OCD and healthy controls. Radhu et al. (66), for instance, found no differences in long intracortical inhibition (LICI) when they compared patients with OCD and control volunteers. LICI is a cortical excitability measure that has also been associated with GABA_B_ receptor functioning (52), but while it is believed to reflect the same neurotransmitter system as CSP, the two measures are methodologically very distinct and probably reflect different neurophysiological processes (67). In fact, LICI reflects change in amplitude of evoked potentials following a conditioning stimulus, whilst CSP represents a temporal dimension measured in milliseconds and is believed to specifically reflect slow postsynaptic GABA_B_ inhibition within the primary motor cortex (M1).

The main limitation of the present study is the reduced number of studies contributing data to the various meta-analyses, meaning that most case-control comparisons were underpowered and potentially biased. This is mostly due to studies often not reporting excitability measures, even when, presumably, they were (some of them, at least) assessed as part of baseline-assessments, for instance in intervention studies. For CSP, which was the only significantly different measure of excitability between the two groups, only five studies provided sufficient meta-analyzable data. As a consequence, we were unable to estimate the effect of relevant confounding factors such as medication effects or psychiatric comorbidity. Some specific SSRIs, among first-line treatments for OCD, have been shown to increase the duration of the CSP (60), and it is thus possible that pooled differences between patients with OCD and control volunteers in this and other cortical excitability measures are under-estimated in the present study, since most patients in the reviewed studies were medicated.

It should be emphasized here that shorter values of CSP are by no means specific to OCD, having also been amply described in patients with Major Depression, who additionally present other changes of intra-cortical inhibition such as lower SICI (30, 68). Controlling for psychiatric co-morbidity, and in particular for co-morbid major depression, is thus of critical importance as patients with OCD enrolled in the eligible studies might have significant psychiatric co-morbidity, reflected in lower values of CSP. However, the fact that the present review found no evidence of reduced SICI in patients with OCD suggests that the shorter CSP is not a reflection of co-morbid major depression and may reflect a specific dysfunction of GABA_B_ mechanisms in OCD, that contrasts with a more generalized abnormality of intracortical GABA neurotransmission present in major depressive disorder. This is further supported by the fact that the only paper in the CSP meta-analysis that reported no group-differences for this measure was also the only study that assessed CSP using a low-intensity test stimulus. CSP assessed with test-stimuli at 120% of the RMT and below are typically considered to reflect GABA_A_ activity, while CSP measured with higher intensity test-stimuli is believed to be more closely dependent on GABA_B_ mechanisms (27, 52).

Another important limitation results from the broad heterogeneity across studies in terms of assessment measures and parameters (Supplementary Table 6). For instance, in the case of CSP, different authors consider different starting points for its measurement, with some including the MEP duration as part of the CSP, while others only consider that the CSP starts at the offset of the MEP. In the case of paired-pulse measurements, such as SICI and ICF, although different ISI’s are often used, results are typically only reported as an average of all the ISI’s rather than separately for each ISI [e.g., (28)]. This variability is further compounded by use of different percentages of the RMT when delivering test and conditioning stimulus, or even by the fact that some studies calibrate stimulus intensity with reference to AMT rather than RMT, resulting in additional layers of heterogeneity that limit comparability even further.

Despite these limitations, the results of our systematic review and meta-analysis of motor cortical excitability in OCD suggest that patients with OCD have higher motor cortical excitability than control subjects, and that such increased excitability might reflect abnormal GABA_B_ receptor-mediated GABAergic activity. It is possible, or even likely, that as the number of published studies continues to grow, other measures of cortical excitability may prove to be significantly different in patients with OCD and control volunteers. In the meantime, our conclusions must remain preliminary, and while our main finding converges with findings from other sources that also report evidence of dysfunctional GABA-mediated neurotransmission, the main practical conclusion of the present review is inevitably that cortical excitability in OCD remains insufficiently explored. Although CSP deficits are not specific of OCD and are unlikely to be useful as a diagnostic biomarker of the disorder, this and other cortical excitability measures might still provide us with viable, easily accessible biomarkers to help predict, at the individual level, clinical response to rTMS and other treatments for OCD. Such biomarkers would surely be a welcome improvement in our therapeutic approach to a disorder that remains notoriously difficult to treat in the majority of cases. To be able to contribute toward this end, future studies that use TMS-EMG paradigms to assess patients with OCD, be it for research purposes or as part of an rTMS treatment protocol, must strive to collect and report as many measures of cortical excitability as possible, and to adopt up-to-date, internationally consensual measurement and stimulation methods and parameters. Only then will we have enough good-quality data to fully clarify whether spinal-cortical excitability is abnormal in OCD, and to fully grasp the potential implications of such abnormalities for the current neurobiological models of this disorder. Moreover, we will then be in a better position to explore whether any such measure of cortical excitability may prove to correlate with the probability of response to rTMS treatment or, for that matter, other forms of treatment for this therapeutically pugnacious disorder. Future studies should also address unanswered questions such as the influence of relevant clinical variables such as psychotropic medication, disease duration, or comorbidity, on cortical excitability measures.

## Data availability statement

The original contributions presented in this study are included in the article/Supplementary material, further inquiries can be directed to the corresponding author.

## Author contributions

DR and JB-C conceived and designed the work. DR and AM acquired the data. DR, AM, GC, AO-M, and JB-C analyzed and interpreted the data. DR drafted the work. AM, GC, JO, AO-M, and JB-C revised the manuscript critically for important intellectual content. All authors approved the final version to be published and agreed to be accountable for all aspects of the work in ensuring that questions related to the accuracy or integrity of any part of the work are appropriately investigated and resolved.
