# Supplementary material for: Motor cortical inhibitory deficits in patients with obsessive-compulsive disorder–A systematic review and meta-analysis of transcranial magnetic stimulation literature
Source: Front Psychiatry. 2022 Dec 7;13:1050480. doi: 10.3389/fpsyt.2022.1050480 (PMC9770010; doi:10.3389/fpsyt.2022.1050480)
Supplement: Supplementary file 1 [file Data_Sheet_1.pdf]

Supplementary material

Supplementary method 1 - Syntax

Pubmed

((("obsessive-compulsive") OR ("obsessive compulsive") OR (OCD) OR (obsession) OR (compulsion) OR (obsessive) OR (compulsive) OR ("anxiety disorder")) AND ((“theta burst stimulation”) OR (tbs) OR (“transcranial magnetic stimulation”) OR (tms) OR (rtms) OR (“repetitive Transcranial Magnetic Stimulation”) OR (“intermittent Theta Burst”) OR (iTBS) OR (“continuous Theta Burst”) OR (cTBS)) AND ((excitation) OR (excitability) OR (modulation) OR (modulate) OR (control) OR (change) OR (modify) OR (activity) OR (activate) OR (deactivate) OR (facilitate) OR (facilitation) OR (inhibit) OR (improve) OR (impair) OR (inhibition) OR (adjust) OR (adjustment) OR (transform) OR (induce) OR (induced) OR (modulated) OR (decrease) OR (affect)))

Filters: Humans; English, Portuguese, Spanish, French, German

Web of Science

TS= ("obsessive-compulsive" OR "obsessive compulsive" OR OCD OR obsession OR compulsion OR obsessive OR compulsive OR "anxiety disorder") AND TS=(“theta burst stimulation” OR tbs OR “transcranial magnetic stimulation” OR tms OR rtms OR “repetitive Transcranial Magnetic Stimulation” OR “intermittent Theta Burst” OR iTBS OR “continuous Theta Burst” OR cTBS) AND TS=(excitation OR excitability OR modulation OR modulate OR control OR change OR modify OR activity OR activate OR deactivate OR facilitate OR facilitation OR inhibit OR improve OR impair OR inhibition OR adjust OR adjustment OR transform OR induce OR induced OR modulated OR decrease OR affect)

Filters: Humans; English, Portuguese, Spanish, French, German

EMBASE

("obsessive-compulsive" OR "obsessive compulsive" OR OCD OR obsession OR compulsion OR obsessive OR compulsive OR "anxiety disorder") AND (“theta burst stimulation” OR tbs OR “transcranial magnetic stimulation” OR tms OR rtms OR “repetitive Transcranial Magnetic Stimulation” OR “intermittent Theta Burst” OR iTBS OR “continuous Theta Burst” OR cTBS) AND (excitation OR excitability OR modulation OR modulate OR control OR change OR modify OR activity OR activate OR deactivate OR facilitate OR facilitation OR inhibit OR improve OR impair OR inhibition OR adjust OR adjustment OR transform OR induce OR induced OR modulated OR decrease OR affect)

Filters: Humans; English, Portuguese, Spanish, French, German

PsycINFO

("obsessive-compulsive" OR "obsessive compulsive" OR OCD OR obsession OR compulsion OR obsessive OR compulsive OR "anxiety disorder") AND (“theta burst stimulation” OR tbs OR “transcranial magnetic stimulation” OR tms OR rtms OR “repetitive Transcranial Magnetic Stimulation” OR “intermittent Theta Burst” OR iTBS OR “continuous Theta Burst” OR cTBS) AND (excitation OR excitability OR modulation OR modulate OR control OR change OR modify OR activity OR activate OR deactivate OR facilitate OR facilitation OR inhibit OR improve OR impair OR inhibition OR adjust OR adjustment OR transform OR induce OR induced OR modulated OR decrease OR affect)

Filters: Humans; English, Portuguese, Spanish, French, German

Supplementary method 2

Mean differences between pre- and post-treatment assessments of cortical excitability, within each group, were calculated based on the following formula:

$$Pre - post_{mean\ difference} = Pre_{mean} - Post_{mean}$$

To compute Hedge’s g effect size, the following formula was used (Borenstein et al, 2009):

$$Hedges's\ g = J \times \frac{Pre - post_{mean\ difference}}{S_{within}}$$

Where *J* is given by

$$J = 1 - \frac{3}{4df - 1}$$

And *df* is the degrees of freedom, calculated as the sample size – 1. S_{within} is the within-groups standard deviation, pooled across groups, given by

$$S_{within} = \frac{S_{diff}}{\sqrt{2(1 - r)}}$$

and S_{diff} is the standard deviation of the difference scores and has the following formula

$$S_{diff} = \sqrt{s_{pre}^2 + s_{pos}^2 - 2 \times r \times S_{pre} \times S_{pos}}$$

S_{pre} *and* S_{pos} are the standard deviations of Pre_{mean} and $Post_{mean}$, respectively, and *r* stands for the correlation between the cortical excitability measures acquired before and after treatment. When authors provided individual data on their studies, *r* was directly computed based on the study’s data. When this information was not available, we used a weighted average of the *r* values obtained from studies for which individual data had been provided (Supplementary method 3).

The standard error of the effects sizes (*SE*) was calculated based on

$$SE_g = \sqrt{V_g}$$

Where

$$Vg = J^2 \times V_d$$

and

$$V_d = \left(\frac{1}{n} + \frac{d^2}{2n}\right) 2(1 - r)$$

d is Cohen's effect size which is given by Hedge's g formula without *J* correction factor and *n* is the sample size. Meta-analyses based on such effect sizes and respective *SE* were computed with *RStudio's metagen* function, using an inverse variance method (Schwarzer, 2007).

Supplementary method 3 – *r* values to compute Hedge's *g* effect size in pre-post study design

	r	**p**	**n**
**Donse, 2017**	0.63	<0.01	17
**Carmi, 2019**	0.96	<0.01	47
**Weighted average used for the remaining study**	0.87		

Supplementary Table 1 – Motor cortical excitability measures definition

**Cortical excitability measure**	**Definition**
Resting Motor Threshold (RMT)	Minimum intensity needed to elicit a visible muscle contraction or an electromyographic event of amplitude equal or superior to 50µV in, at least, 5 out of 10 pulses.
Active Motor Threshold (AMT)	Minimum intensity needed to elicit a visible muscle contraction or an electromyographic event of amplitude equal or superior to 200µV in, at least, 5 out of 10 pulses, whilst the person is performing a mild muscle contraction.
Cortical Silent Period (CSP)	Reflects the duration of the period of suppression in electromyographic activity that typically follows a single TMS pulse delivered to the primary motor cortex while a mild voluntary contraction of the corresponding muscle of interest is being performed.
Motor Evoked Potentials (MEP)	Motor evoked potentials are the electrical signals recorded from muscles following stimulation of motor pathways within the brain.
120/140 Ratio	Motor evoked potential amplitude ratio at increasing stimulus intensity from 140% to 120%.
Intracortical Facilitation (ICF)	Increase in cortical excitability evoked by conditioning stimuli and assessed by test stimuli in a conditioning-test paradigm, with a specific interval range between the stimuli (6-25ms).
Short-interval Intracortical Inhibition (SICI)	Decrease in cortical excitability evoked by conditioning stimuli and assessed by test stimuli in a conditioning-test paradigm, with a specific interval range between the stimuli (1-6ms).
Long-Interval Intra-cortical Inhibition (LICI)	Decrease in cortical excitability evoked by conditioning stimuli and assessed by test stimuli in a conditioning-test paradigm, with a specific interval range between the stimuli (50-200ms).
Cortical excitability modulation measures	Ratio and/or difference between all aforementioned excitability measures before and after a single session of repetitive transcranial magnetic stimulation (rTMS) or theta burst stimulation (TBS) or other excitability modulation protocol applied to motor cortex.

Supplementary Table 2 – Motor cortical excitability measures data

	Resting Motor Threshold (%)		Active Motor Threshold (%)		MEP amplitude (mV)		Intracortical Inhibition (%)		Intracortical Facilitation (%)		Cortical Silent Period (ms)	
	**OCD (mean ± SD)**	**CV (mean ± SD)**	**OCD (mean ± SD)**	**CV (mean ± SD)**	**OCD (mean ± SD)**	**CV (mean ± SD)**	**OCD (mean ± SD)**	**CV (mean ± SD)**	**OCD (mean ± SD)**	**CV (mean ± SD)**	**OCD (mean ± SD)**	**CV (mean ± SD)**
**Badawy 2010**	68.63±9.15	-	-	-	-	-	-	-	-	-	-	-
**Greenberg 2000**	42.40±6.80	51.90±8.00	33.10±3.80	42.60±7.80	-	-	93.00±128.00	56.00±107.00	121.00±123.00	113.00±143.00	88.00±37.49	85.15±26.27
**Hedge 2016**	36.21±12.29	-	-	-	-	-	-	-	-	-	-	-
**Kang 2009**	51.20±13.53	-	-	-	-	-	-	-	-	-	-	-
**Kang 2019**	46.75±11.93	47.64±10.93	-	-	-	-	61.00±59.00	48.00±41.00	113.00±79.00	170.00±75.00	150.92±24.93	167.77±29.48
**Khedr 2016**	38.18±4.47	47.10±8.10	29.13±3.85	36.10±8.90	1.49±0.39	1.10±0.20	43.34±22.46	53.50±20.03	124.37±47.57	133.70±39.90	105.80±24.39	152.30±21.70
**Mantovani 2006**	65.80±9.60	-	-	-	-	-	-	-	-	-	-	-
**Mantovani 2013**	-	-	37.85±7.51	-	-	-	-	-	-	-	-	-
**Richter 2012**	45.63±9.83	44.85±8.56	-	-	0.98±0.33	0.90±0.27	73.60±55.00	56.50±34.00	161.00±66.00	128.00±42.00	122.00±36.30	153.50±32.00
**Suppa 2014**	37.35±6.55	36.75±6.60	49.80±10.10	42.90±7.95	-	-	-	-	-	-	-	-
**Donse 2017**	52.82±13.86	-	-	-	-	-	-	-	-	-	-	-
**Harika 2019**	48.04±10.12	-	-	-	-	-	-	-	-	-	-	-
**Carmi, 2019**	62.81±10.52	-	-	-	-	-	-	-	-	-	-	-
**Elbeh, 2016**	38.25±5.21	-	29.25±4.19	-	-	-	-	-	-	-	-	-
**de Wit, 2015**	48.46±7.67	48.34±7.06	-	-	-	-	-	-	-	-	-	-
**Metha, 2021**	31.90±6.32	35.20±6.22	-	-	-	-	24.00±29.10	27.20±38.00	-	-	76.8±41.50	106.0±41.80
**Russo, 2014**	36.30±1.00	44.20±2.80	-	-	-	-	109.00±35.00	45.00±7.00	143.00±69.00	141.00±69.00	-	-

Legend: MEP – motor evoked potentials; OCD – obsessive-compulsive disorder; CV – control volunteers; mV – millivolts; SD – standard deviation

Supplementary Table 3 – Study quality assessment by Newcastle-Ottawa Quality Assessment Scale for case-control studies.

Authors, year	Selection															Comparability				Exposure														Total
	1				2			3				4			Sub-total	1			Sub-total	1						2			3				Sub-total	
	a)	b)	c)	Score	a)	b)	Score	a)	b)	c)	Score	a)	b)	Score		a)	b)	Score		a)	b)	c)	d)	e)	Score	a)	b)	Score	a)	b)	c)	Score		
Greenberg 2000	1	-	-	1	-	1	0	-	-	1	0	-	1	0	1	-	-	0	0	1	-	-	-	-	1	1	-	1	1	-	-	1	3	4
Kang 2019	1	-	-	1	-	1	0	1	-	-	1	1	-	1	3	1	-	1	1	1	-	-	-	-	1	1	-	1	1	-	-	1	3	7
Khedr 2016	1	-	-	1	1	-	1	-	-	1	0	1	-	1	3	1	1	2	2	1	-	-	-	-	1	1	-	1	1	-	-	1	3	8
Richter 2012	1	-	-	1	-	1	0	-	-	1	0	1	-	1	2	-	1	1	1	1	-	-	-	-	1	1	-	1	1	-	-	1	3	6
Suppa 2014	1	-	-	1	-	1	0	-	-	1	0	-	1	0	1	-	-	0	0	1	-	-	-	-	1	1	-	1	1	-	-	1	3	4
deWit 2015	1	-	-	1	-	1	0	1	-	-	1	-	1	0	2	1	-	1	1	1	-	-	-	-	1	1	-	1	1	-	-	1	3	6
Mehta, 2021	1	-	-	1	1	-	1	1	-	-	1	1	-	1	4	1	1	2	2	1	-	-	-	-	1	1	-	1	1	-	-	1	3	9
Russo, 2014	-	1	-	0	-	1	0	-	-	1	0	-	1	0	0	-	-	0	0	1	-	-	-	-	1	1	-	1	1	-	-	1	3	3

Selection

1) Is the case definition adequate?
   a) yes, with independent validation **
   b) yes, eg record linkage or based on self reports
   c) no description

2) Representativeness of the cases
   a) consecutive or obviously representative series of cases **
   b) potential for selection biases or not stated

3) Selection of Controls
   a) community controls **
   b) hospital controls
   c) no description

4) Definition of Controls
   a) no history of disease (endpoint) **
   b) no description of source

Comparability

1) Comparability of cases and controls on the basis of the design or analysis
   a) study controls for _______________ (Select the most important factor.) **
   b) study controls for any additional factor ** (This criteria could be modified to indicate specific control for a second important factor.)

Exposure

1) Ascertainment of exposure
   a) secure record (eg surgical records) **
   b) structured interview where blind to case/control status **
   c) interview not blinded to case/control status
   d) written self report or medical record only
   e) no description

2) Same method of ascertainment for cases and controls
   a) yes **
   b) no

3) Non-Response rate
   a) same rate for both groups **
   b) non respondents described
   c) rate different and no designation

Supplementary Table 4– Data regarding cortical excitability measurements that were not meta-analyzed

**Active Motor Threshold – Obsessive compulsive-disorder vs Control volunteers**		
**Study (Author, year)**	**Effect size (*Hedge's g*)**	**Confidence interval (95% CI)**
Greenberg, 2000	-1.60	[-2.50; -0.71]
Khedr, 2016	-1.25	[-1.88; -0.62]
Suppa, 2014	0.74	[0.05; 1.44]
Negative values favor active motor threshold in control volunteers; Positive values favor active motor threshold in patients with obsessive-compulsive disorder		
**Motor Evoked Potential amplitude - Obsessive compulsive-disorder vs Control volunteers**		
**Study (Author, year)**	**Effect size (*Hedge's g*)**	**Confidence interval (95% CI)**
Khedr, 2016	1.09	[0.47; 1.71]
Richter, 2012	0.28	[-0.20; 0.76]
Negative values favor motor evoked potential amplitude in control volunteers; Positive values favor motor evoked potential amplitude in patients with obsessive-compulsive disorder		
**Pre-to-post treatment RMT changes in sham-controlled trials that reported a statistically superior clinical effect of rTMS when compared to sham**		
**Study (Author, year)**	**Effect size (*Hedge's g*)**	**Confidence interval (95% CI)**
Badawy, 2010	-0.77	[-4.20; 2.66]
Carmi, 2019	-0.09	[-0.33; 0.15]
Negative values reflect higher resting motor threshold values after treatment; Positive values reflect higher resting motor threshold values before treatment		
**Interhemispheric asymmetry in cortical excitability as measured by RMT among patients with OCD**		
**Study (Author, year)**	**Effect size (*Hedge's g*)**	**Confidence interval (95% CI)**
Kang, 2019	0.06	[-22.77; 22.89]
Mantovani, 2006	9.80	[-11.88; 31.48]
Negative values reflect higher resting motor threshold values on the right hemisphere; Positive values reflect higher resting motor threshold values on the left hemisphere		

Supplementary Table 5 – Treatment parameters used in trials intended to asses rTMS efficacy for patients with obsessive-compulsive disorder

	**Frequency (Hz)**	**Intensity (RMT %)**	**Number of sessions**	**Number of trains**	**Train duration**	**Interval between trains**	**Pulses per train**	**Pulses per session**	**Target region**	**TMS modality**
**Badawy, 2010**	20		15						Left Dorsolateral Prefrontal Cortex	high-frequency rTMS
**Mantovani, 2006**	1	100	10	4	300s	120s	300	1200	bilateral Supplementary motor area	low-frequency rTMS
**Mantovani, 2013**	1	100	20	1	1200		1200	1200	Supplementary motor area	low frequency
**Donse, 2017**	1	110/100/120	Variable	1	1000s/1200s		1000/1200	1000/1200	Supplementary motor area /right DLPFC for comorbid MDD	low-frequency
**Carmi, 2019**	20	100	29	50	2s	20s	40	2000	Medial pre-frontal cortex-Anterior cingulate cortex (bilaterally)	High-frequency deep TMS

Legend: Hz – Hertz; RMT – resting motor threshold; TMS – transcranial magnetic stimulation; rTMS – repetitive TMS; DLPFC – dorsolateral prefrontal cortex; MDD – major depressive disorder.

Supplementary Figure 1 – Forest plot of changes in resting motor threshold (RMT) values from pre- to- post treatment assessment, in patients with OCD

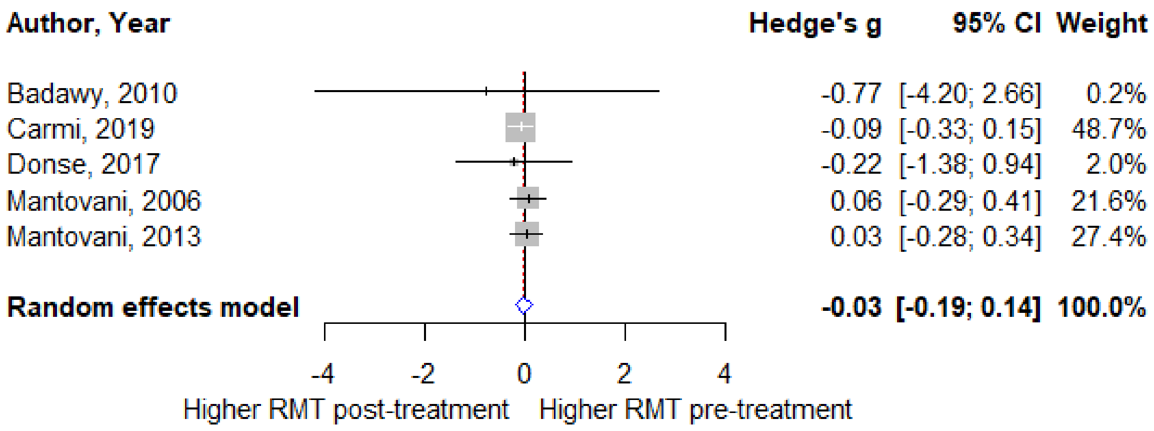

Supplementary Table 6 – Resting Motor Threshold (machine's maximum output - %) data pre- and post-treatment

	**Pre-treatment**		**Post-treatment**		**Clinical efficacy of rTMS treatment in Randomized controlled trials** (Intervention statistically superior to sham?)
	**Mean**	**Standard deviation**	**Mean**	**Standard deviation**	
**Badawy, 2010**	67.50	9.10	74.80	9.50	Yes
**Carmi, 2019**	62.80	10.40	63.70	10.30	Yes
**Donse, 2019**	52.80	13.90	56.10	14.10	No
**Mantovani, 2006**	65.80	9.60	64.80	13.50	n/a
**Mantovani, 2013**	47.00	10.70	46.50	13.60	No

Supplementary Figure 2 – Forest plot comparing cortical silent period (CSP) values between patients with obsessive-compulsive disorder and control volunteers, considering only studies with a score superior to 5 in the Newcastle-Ottawa Quality Assessment Scale.

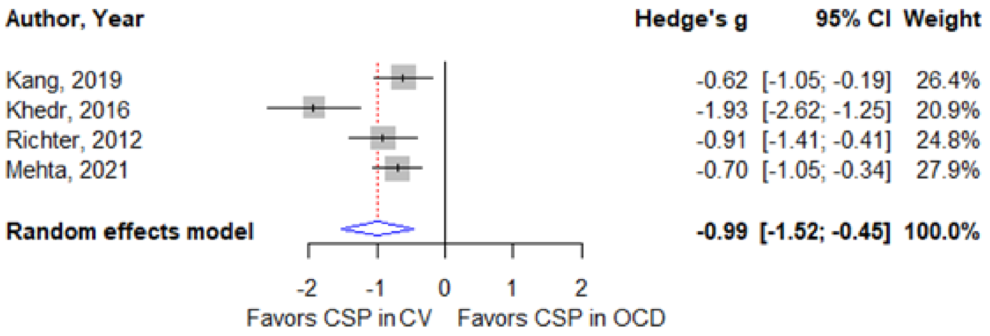

Supplementary Table 7 - Methods used to acquire motor cortical excitability measures in each case-control study

	**Muscle used for assessment**	**RMT**	**AMT**	**MEP**	**ICF**	**SICI**	**CSP**
**de Wit et al, 2015**	Not mentioned	Not described					
**Greenberg *et al*, 2000**	APB	At least 5 out of 10 pulses generating an MEP of 50 µV or larger	At least 5 out of 10 pulses generating an MEP of 200 µV or larger		CS applied at 95% of the AMT; TS applied to generate an MEP of around 500 µV to 1000 µV of amplitude<br><br>ISI's of 7,8,9,10,15,20 ms	CS applied at 95% of the AMT; TS applied to generate an MEP of around 500 µV to 1000 µV of amplitude<br><br>ISI's of 2,3,4,5,6 ms	TMS stimulus applied at 110% and 120% of the RMT while with muscle "mildly" contracted<br><br>CSP is measured starting at the offset of the MEP and ends when basal myographic activity returns.
**Kang et al, 2019**	ADM	At least 5 out of 10 pulses generating an MEP of 50 µV or larger			CS applied at 80% of the RMT; TS applied to generate an MEP of around to 1000 µV of amplitude<br><br>ISI's of 10, 15 ms	CS applied at 80% of the RMT; TS applied to generate an MEP of around to 1000 µV of amplitude<br><br>ISI's of 2, 3 ms	TMS stimulus applied at 140% the RMT while with a "low-level voluntary" muscle contraction<br><br>CSP is measured starting at the offset of the MEP and ends when basal myographic activity returns.

Legend: APB – *abductor pollicis brevis*; ADM - *abductor digiti minimi*; FDI – First Dorsal Interosseous; TS – Test stimulus; CS – Conditioning stimulus; ISI – Interstimulus interval; TMS – Transcranial magnetic stimulation; RMT – Resting motor threshold; AMT – Active motor threshold; MEP – Motor Evoked Potental; ICF- Intracortical facilitation; SICI – Short intracortical inhibition; CSP – Cortical silent period; µV – microvolts; ms - milliseconds

Supplementary Table 7 (Continuation)- Methods used to acquire motor cortical excitability measures in each case-control study

**Khedr et al, 2016**	FDI	At least 5 out of 10 pulses generating an MEP of 50 µV or larger	At least 5 out of 10 pulses generating an MEP of 200 µV or larger	Measurement of Motor Evoked potentials peak-to-peak amplitude.	CS applied at 80% of the RMT; TS applied to generate an MEP of around to 1000 µV of amplitude<br><br>ISI's of 8, 10, 12 ms	CS applied at 80% of the RMT; TS applied to generate an MEP of around to 1000 µV of amplitude<br><br>ISI's of 2, 3, 4 ms	TMS stimulus applied at 130% while the muscle is at 50% of a maximum voluntary contraction<br><br>CSP is measured starting at the offset of the MEP and ends when basal myographic activity returns.
**Mehta et al, 2021**	FDI	At least 5 out of 10 pulses generating an MEP of 50 µV or larger		Measurement of Motor Evoked potentials peak-to-peak amplitude.<br><br>Reports results in percentage of the machine's maximum output to obtain, on average, MEP's of approximately 1mV.		CS applied at 80% of the RMT; TS applied to generate an MEP of around to 1000 µV of amplitude<br><br>ISI's of 3 ms	Suprathreshold TMS pulse while with a "tonically active muscle"<br><br>CSP is measured starting at the offset of the MEP and ends when basal myographic activity returns.

Legend: APB – Abductor Pollicis Brevis; ADM - Abductor digiti minimi; FDI – First Dorsal Interosseous; TS – Test stimulus; CS – Conditioning stimulus; ISI – Interstimulus interval; TMS – Transcranial magnetic stimulation; RMT – Resting motor threshold; AMT – Active motor threshold; MEP – Motor Evoked Potental; ICF- Intracortical facilitation; SICI – Short intracortical inhibition; CSP – Cortical silent period; µV – microvolts; ms - milliseconds

Supplementary Table 7 (Continuation)- Methods used to acquire motor cortical excitability measures in each case-control study, for each extracted measure

**Richter *et al*, 2012**	APB	At least 5 out of 10 pulses generating an MEP of 50 µV or larger		Measurement of Motor Evoked potentials peak-to-peak amplitude.	CS applied at 80% of the RMT; TS applied to generate an MEP of around to 1000 µV of amplitude<br><br>ISI's of 10, 12, 15 ms	CS applied at 80% of the RMT; TS applied to generate an MEP of around to 1000 µV of amplitude<br><br>ISI's of 2, 4 ms	TMS stimulus applied at 140% the RMT while with a muscle at 20% of a maximum voluntary contraction<br><br>CSP is measured starting at the onset of the MEP and ends when basal myographic activity returns.
**Russo et al, 2014**	APB	At least 5 out of 10 pulses generating an MEP of 50 µV or larger			CS applied at 80% of the AMT; TS applied to generate an MEP of around to 1000 µV of amplitude<br><br>ISI's of 10	CS applied at 80% of the AMT; TS applied to generate an MEP of around to 1000 µV of amplitude<br><br>ISI's of 2 ms	
**Suppa *et al*, 2014**	FDI	*"Increase the stimulus intensity progressively at 5% steps until reaching a level which induces reliable (usually around 100 µV) MEPs in about 50% of 10-20 consecutive stimuli and define this as the excitability threshold." (Rossini et al, 1998*	Not described				

Legend: APB – Abductor Pollicis Brevis; ADM - Abductor digiti minimi; FDI – First Dorsal Interosseous; TS – Test stimulus; CS – Conditioning stimulus; ISI – Interstimulus interval; TMS – Transcranial magnetic stimulation; RMT – Resting motor threshold; AMT – Active motor threshold; MEP – Motor Evoked Potental; ICF- Intracortical facilitation; SICI – Short intracortical inhibition; CSP – Cortical silent period; µV – microvolts; ms - milliseconds

Supplementary Figure 3 - Forest plot comparing resting motor threshold (RMT) values, between patients with obsessive-compulsive disorder and control volunteers, considering only studies with the same acquisition methodology

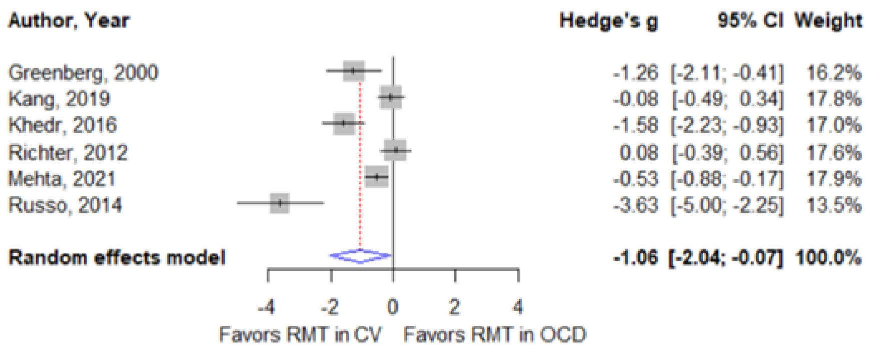

References Supplementary materials

1. Rossini, P. M., Barker, A. T., Berardelli, A., Caramia, M. D., Caruso, G., Cracco, R. Q., … Tomberg, C. (1994). Non-invasive electrical and magnetic stimulation of the brain, spinal cord and roots: basic principles and procedures for routine clinical application. Report of an IFCN committee. Electroencephalography and Clinical Neurophysiology, 91(2), 79–92. doi:10.1016/0013-4694(94)90029-9

2. Borenstein, M., Hedges, L. V., Higgins, J. P. T., & Rothstein, H. (2009). *Introduction to meta-analysis*. Chichester, U.K: John Wiley & Sons.

3. Schwarzer G (2007): meta: An R package for meta-analysis. R News, 7, 40–5
